# Atrial Fibrillation Is Associated with a Marker of Endothelial Function and Oxidative Stress in Patients with Acute Myocardial Infarction

**DOI:** 10.1371/journal.pone.0131439

**Published:** 2015-07-09

**Authors:** Karim Stamboul, Julie Lorin, Luc Lorgis, Charles Guenancia, Jean-Claude Beer, Claude Touzery, Luc Rochette, Catherine Vergely, Yves Cottin, Marianne Zeller

**Affiliations:** 1 Cardiology Department, University Hospital, Dijon, France; 2 Laboratory of Cardiometabolic Physiopathology and Pharmacology, UMR INSERM U866, University of Burgundy, Dijon, France; University Hospital Medical Centre, GERMANY

## Abstract

**Background:**

Atrial fibrillation (AF), whether silent or symptomatic, is a frequent and severe complication of acute myocardial infarction (AMI). Asymmetric dimethylarginine (ADMA), an endogenous eNOS inhibitor, is a risk factor for endothelial dysfunction. We addressed the relationship between ADMA plasma levels and AF occurrence in AMI.

**Methods:**

273 patients hospitalized for AMI were included. Continuous electrocardiographic monitoring (CEM) ≥48 hours was recorded and ADMA was measured by High Performance Liquid Chromatography on admission blood sample.

**Results:**

The incidence of silent and symptomatic AF was 39(14%) and 29 (11%), respectively. AF patients were markedly older than patients without AF (≈ 20 y). There was a trend towards higher ADMA levels in patients with symptomatic AF than in patients with silent AF or no AF (0.53 vs 0.49 and 0.49 μmol/L, respectively, p = 0.18,). After matching on age, we found that patients with symptomatic AF had a higher heart rate on admission and a higher rate of patients with LV dysfunction (28% vs. 3%, p = 0.025). Patients who developed symptomatic AF had a higher ADMA level than patients without AF (0.53 vs. 0.43 μmol/L; p = 0.001). Multivariate logistic regression analysis to estimate symptomatic AF occurrence showed that ADMA was independently associated with symptomatic AF (OR: 2.46 [1.21–5.00], p = 0.013) beyond history of AF, LVEF<40% and elevated HR.

**Conclusion:**

We show that high ADMA level is associated with the occurrence of AF. Although no causative role can be concluded from our observational study, our work further supports the hypothesis that endothelial dysfunction is involved in the pathogenesis of AF in AMI.

## Introduction

Atrial fibrillation (AF) is a major public health burden worldwide, and its prevalence is set to increase owing to widespread population ageing [1]. AF, either subclinical (i.e. silent) or symptomatic, often complicates acute myocardial infarction (AMI), with an incidence ranging from 6 to 21% [2, 3,4]^.^ Both silent and symptomatic AF have serious adverse prognostic implications across the whole spectrum of acute coronary syndromes for in-hospital and long-term mortality [2, 3, 5]. Reduced Nitric Oxide (NO) availability and subsequent endothelial dysfunction have recently been recognized as possible contributors to the worse prognosis in AF [6]. Moreover, NOS inhibitors are a new challenging therapeutic target in coronary artery disease (CAD) [7].

Dimethylarginines, including Asymmetric dimethylarginine (ADMA) and Symmetric dimethylarginine (SDMA), are endogenous methylated analogues of L-arginine, the precursor of NO. ADMA can inhibit NO synthase (NOS) and thus causes endothelial dysfunction, inflammation and oxidative stress in cardiovascular diseases [8, 9]. Symmetric dimethylarginine (SDMA) does not inhibit NOS directly but could interfere with the cellular uptake of L-arginine [10]. At levels encountered in patients with CAD, ADMA experimentally impairs endothelium-dependent relaxation and is considered a circulating marker of endothelial dysfunction [11]. ADMA also causes cardiac dysfunction with decreased cardiac output and increased vascular resistance, and causes vascular injury in animal models [12–14]. Moreover, a high plasma ADMA level has been reported to be an independent risk factor for adverse cardiovascular events and mortality in patients with CAD [15, 16].

Interestingly, elevated circulating levels of ADMA have been found in patients with chronic or acute AF and are predictors of recurrence after electrical cardioversion [17–19]. Moreover, a recent study showed that AF patients referred for a coronary angiogram had a higher level of ADMA than no-AF patients, and experienced more cardiovascular (CV) events in the long term, independently of current risk scores [20]. However, no study has addressed the relationship between ADMA levels and the occurrence of AF in AMI.

The purpose of our study was to determine whether the methylated derivatives of L-arginine (ADMA and SDMA) could be related to the occurrence of AF in patients hospitalized for AMI.

## Methods

### Patients

From 1^st^ May 2011 to 31^st^ January 2013, all consecutive patients hospitalized for AMI at the Coronary Care Unit of Dijon University Hospital who underwent continuous electrographic monitoring (CEM) were included, with the following criteria: 1) age≥18 years, and 2) CEM≥48 hours. Patients with cardiogenic shock or who underwent CABG were excluded from the study.

MI was defined by an increase in serum troponin I (2X. upper limit of the hospital normal (ULN) range) associated with symptoms of ischemia and/or characteristic ECG signs (ST segment or T wave changes, left bundle branch block, or the development of pathological Q waves). STEMI was defined as chest pain lasting for ≥20min with typical ECG changes including ≥1mV ST-segment elevation in two or more limb leads or ≥2mV in two or more contiguous precordial leads.

Cardiovascular history, current medications, risk factors, clinical data, acute management and in-hospital clinical outcomes (including death, stroke, and heart failure) were recorded. Heart failure was defined by Killip class >1. The independent ethics committee of Dijon University Hospital approved the study protocol and written informed consent was obtained from all patients or their legal representatives.

### Biological data

Blood samples were drawn on admission. The median (interquartile range, IQR) time from symptom onset to blood sampling was 17(8–28) hours. Plasma N-terminal fragment of B-type natriuretic peptide (Nt-proBNP) was measured using homogeneous chemiluminescence immunoassay on a Vista analyzer (SIEMENS). Blood glucose was assessed by the enzymatic method on a Vitros 950 analyzer (Ortho Clinical Diagnostics, Rochester, NY). Plasma creatinine levels were measured on a Vitros 950 analyzer (Ortho Clinical Diagnostics, Rochester, NY). Glomerular Filtration Rate was calculated by CKD-EPI formula [21]. C-reactive protein (CRP) was determined on a dimension Xpand (Dade Behring, Deerfield, IL) using enzymatic methods. The CRP level was dichotomized at 3 mg/L for more clinical relevance.

### Dimethylarginines and L-arginine measurement

Samples were allowed to clot at room temperature for 30 minutes and centrifuged at 2500 rpm for 10 minutes at 4°C. The serum was kept frozen at -80°C until analysis. L-arginine, ADMA and SDMA, were measured by high performance liquid chromatography (HPLC) as previously described [22, 23]. Assays were performed in duplicate.

### Echocardiography

The Left Ventricular Ejection Fraction (LVEF) and Left Atrial (LA) dimensions were assessed by echocardiography within 48h. LVEF was calculated using the Simpson method at the apical four-chamber (A4C) and apical two-chamber (A2C) views. LVEF was dichotomized at 40% for more clinical relevance. LA volume was calculated using the bi-plane area-length method at the A4C view at ventricular end systole (maximum LA size) and was indexed to body surface area (BSA).

### Atrial fibrillation

All of the patients who met the inclusion criteria had undergone both CEM and echocardiography. AF was diagnosed in accordance with the current European Society of Cardiology Guidelines as absolutely irregular RR intervals, no distinct P waves and atrial cycle length (when visible) ≤ 200ms (atrial rate ≥ 300bpm) [24].

CEM, using Philips intellvue MP50, was started immediately after hospital admission and continued during the first 48 hours thereafter as previously described^4^. Symptomatic AF was defined as AF occurring within 48H after admission, resulting in clinical symptoms or the need for urgent cardioversion and confirmed on the ECG. Silent AF was defined as the occurrence of at least one episode of asymptomatic AF, and detected by automatic CEM. Symptomatic AF was diagnosed using routine screening methods (12-lead ECG and clinical examination, with interview of the patient, but no questionnaire). Patients who experienced successive episodes of silent and symptomatic AF during the monitoring period were classified as having symptomatic AF. The automatic diagnosis of SVES was based on the detection of a range of 300 bpm, with a resolution of 1 bpm. The study population was divided into three groups: silent AF, symptomatic AF and no AF.

### Statistical analysis

Data are presented as medians (IQR), means±SD or proportions (n(%)). For continuous variables, normality was checked by the Kolmogorov-Smirnov test. Student t test was used for 2-group comparisons of normally distributed data and Mann-Whitney rank sum test for skewed data. Kruskal-Wallis one-way analysis of variance by rank, for non-normally distributed values or one-way ANOVA for normally distributed values was used for 3-group comparisons. Categorical variables were compared by the chi-square test or Fisher’s exact test.

The independent factors associated with symptomatic AF were tested by logistic regression analyses. Variables entered into the multivariate model (model 1) were chosen based on their significant relationship (p<0.10) in univariate analysis, i.e. prior AF, LVEF<40%, and HR on admission. A second model (model 2) was built with covariates from model 1+ ADMA. The goodness of the fit was tested by the -2Log Likelihood χ² criteria as an index of model quality. The additional prognostic information of ADMA was tested by comparing the -2log likelihoods of model 2 vs model 1 and by category-free Net Reclassification Improvement (NRI). Statistical analyses were performed using SPSS 12.0 software (IBM Inc, USA).

## Results

### Characteristics

The incidence of silent or symptomatic AF post MI, was 39(14%) and 29 (11%), respectively (n = 273) (Table 1). When compared to the No-AF group, silent and symptomatic AF patients were almost 20 y older (63 vs. 80 and 82y, respectively), less likely to be smokers and more frequently hypertensive. They were more likely to have a CV history, including prior CAD, AF and renal failure. AF patients more frequently received chronic CV medications, such as statins, angiotensin converting enzyme (ACE) inhibitor, Beta-blocker and aspirin than did No-AF patients. For acute therapies, AF patients received less Beta-blockers, ACE inhibitors (24 vs. 54%) and conversely more diuretics (55 vs. 17%) and amiodarone (45 vs. 4%) than did No-AF patients. AF patients were also less likely to be treated by PCI (41 vs. 69%). AF patients had a higher HR on admission, more heart failure, and had more frequently LV dysfunction, with a longer ICU stay. On admission, patients in the AF groups had a lower eGFR. Nt-proBNP levels gradually increased across the 3 groups. L-arginine level was similar for the 3 groups. However, there was a trend towards higher ADMA levels in patients with symptomatic AF than in patients with silent AF or no AF (0.53 vs 0.49 and 0.49 μmol/L, respectively). SDMA serum levels gradually increased across the 3 groups (p = 0.005). LA volume indexed gradually increased across the 3 groups, and AF groups had significant LA enlargement compared with patients without AF (Table 1).

**Table 1:** Patients’ characteristics (n(%), median (IQR)).

	No AF N = 205	Silent AF N = 39	Symptomatic AF N = 29	p
**Risk factors**				
Age, *years*	63 [53–76]	80 [64–85]*	82 [74–89]‡	<0.001
Female	63 (31%)	16 (41%)	11 (38%)	0.380
BMI, *kg/m^2^*	26 [24–29]	26 [24–30]	27 [23–30]	0.848
Hypertension	92 (45%)	26 (67%)*	21 (72%)‡	0.002
Hypercholesterolemia	87 (42%)	21 (54%)	12 (41%)	0.403
Family history of CAD	77 (38%)	8 (21%)*	6 (21%)	0.037
Diabetes	47 (23%)	12 (31%)	6 (21%)	0.526
Smoking	70 (34%)	7 (18%)*	3 (10%)‡	0.008
**CV history**				
CAD	29 (14%)	11 (28%)*	11 (38%)‡	0.002
Stroke	5 (2%)	2 (5%)	2 (7%)	0.178
Chronic renal failure	6 (3%)	4 (10%)	5 (17%)‡	0.002
Atrial fibrillation	5 (2%)	2 (5%)	6 (21%)‡	0.001
**Chronic medications**				
Aspirin	33 (16%)	14 (36%)*	10 (35%)‡	0.003
Beta-Blockers	43 (21%)	16 (41%)*	13 (45%)‡	0.002
ACE inhibitor	27 (13%)	6 (15%)	10 (35%)‡	0.023
Diuretic	38 (19%)	18 (46%)*	9 (31%)	0.001
Statin	49 (24%)	17 (44%)*	12 (41%)‡	0.012
VKA	8 (4%)	5 (13%)*	3 (10%)	0.044
Digoxin	1 (1%)	1 (3%)	1 (3%)	0.154
Amiodarone	4 (2%)	0 (0%)	0 (0%)	1
**Acute therapies**				
Aspirin	202 (99%)	39 (100%)	28 (97%)	0.441
Beta-Blockers	169 (82%)	27 (69%)	17 (59%)‡	0.005
ACE inhibitor	110 (54%)	11 (28%)*	7 (24%)‡	<0.001
Diuretic	35 (17%)	13 (33%)*	16 (55%)‡	<0.001
Statin	198 (97%)	37 (95%)	28 (97%)	0.858
VKA	5 (2%)	2 (5%)	3 (10%)	0.058
Digoxin	1 (1%)	0 (0%)	1 (3%)	0.221
Amiodarone	9 (4%)	1 (3%)†	13 (45%)‡	<0.001
PCI	141 (69%)	24 (62%)	12 (41%)‡	0.014
**Clinical data**				
HR, *beats/min*	75 [66–87]	75 [63–89]†	93 [77–102] ‡	0.001
SBP, *mmHg*	141 ± 27	134 ± 28	130 ± 26‡	0.049
DBP, *mmHg*	81 ± 18	77 ± 17	77 ± 16	0.133
Heart failure	33 (16%)	10 (26%)†	16 (55%)‡	<0.001
Anterior wall location	68 (33%)	15 (39%)	12 (41%)	0.599
STEMI	101 (49%)	22 (56%)	10 (35%)	0.192
Symptom onset to admission, *min*	180 [100–391]	180 [121–518]	300 [148–735]	0.219
ICU stay, *days*	4 [3–5]	4 [3–5]	5 [4–7] ‡	0.017
LVEF (%)	55 [45–60]	55 [40–60]†	40 [35–55] ‡	<0.001
LVEF<40%	16 (8%)	9 (23%)*	8 (28%)‡	0.001
LA Volume indexed (mL/m²)	23.6 [18.1–33.3]	29.5 [21.4–39.7] *	33.9 [25.4–48.2] ‡	<0.001
**Biological data**				
CRP≥3, *mg/L*	132 (64%)	25 (64%)	20 (69%)	0.885
eGFR *ml/min*	78.0 ± 24.1	67.3 ± 26.2*	56.8 ± 23.4‡	<0.001
Glucose, *mmol/L*	6.57 [5.53–8.30]	7.14 [6.20–8.57]	7.09 [5.93–9.31]	0.063
Nt-proBNP, *pg/mL*	471(150–1966)	1632(227–5611) *	4929(1144–18412) ‡	<0.001
L-arginine, *μmol/L*	85.27 [66.29–110.77]	74.72 [65.66–102.59]	75.84 [54.52–91.53]	0.090
ADMA, *μmol/L*	0.49 [0.43–0.58]	0.49 [0.40–0.59]	0.53 [0.46–0.63]	0.179
SDMA, *μmol/L*	0.51 [0.43–0.67]	0.63 [0.43–0.83]	0.76 [0.51–1.00] ‡	0.005

ACE: Angiotensin Converting Enzyme; ADMA: Asymmetric Dimethyl Arginine; AF: Atrial fibrillation; BMI: Body mass index; CAD: Coronary artery disease; CKD-EPI: Chronic Kidney Disease-Epidemiology Collaboration; CRP: C-reactive protein; CV: Cardiovascular; DBP: Diastolic blood pressure; eGFR: Estimated Glomerular Filtration Rate; HR: heart rate; ICU: Intensive Care Unit; LA: Left atrial; LVEF: Left ventricular ejection fraction; Nt-proBNP: N-terminal fragment of B-type natriuretic peptide; PCI: Percutaneous Coronary Intervention; SBP: Systolic blood pressure; SDMA: Symmetric Dimethyl Arginine.; STEMI: ST-segment elevation Myocardial Infarction; VKA: Vitamin K Antagonist.

* p<0.05

† No AF versus Silent AF

‡ Silent AF versus Symptomatic AF and No AF versus Symptomatic AF

### In-hospital outcomes analysis

Patients with silent or symptomatic AF were more likely to experience acute heart failure episodes during their hospital stay than the No-AF group (10(26%) and 18(62%) vs. 33(16%), respectively p<0.001). Only 1 patient (from the symptomatic AF group) experienced stroke. Seventeen patients died from cardiovascular causes, and most were from the AF groups, showing that AF patients were at very high risk (Symptomatic AF: 10(35%), silent AF: 3(7.7%) and no AF: 4(2.0%), p<0.001).

### Matched population analysis

**Fig 1:**
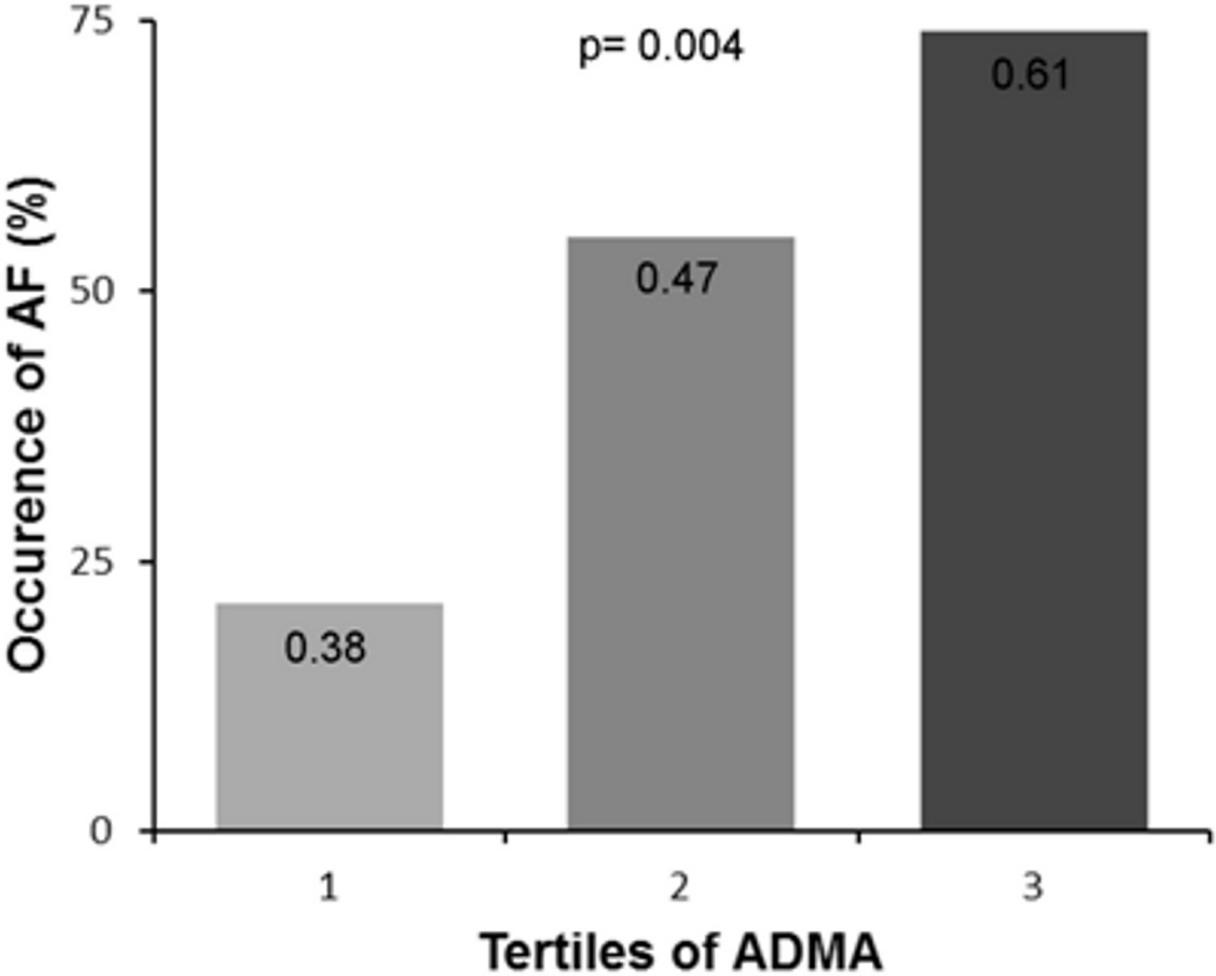
Symptomatic AF occurrence in tertiles of ADMA (p = 0.004). ADMA: Asymetric dimethyl arginine; AF: Atrial Fibrillation.

As symptomatic AF patients were markedly (≈ 20 y) older than No-AF patients, we further attempted to reduce the bias of age and related covariables by nearest-neighbor matching. Therefore, the 29 patients who developed symptomatic AF were matched for age with 29 patients from the No-AF group. After matching, we found no difference regarding CV risk factors, history and chronic medications in the 2 groups (Table 2). Patients with symptomatic AF had a higher HR on admission (90 vs. 77 beats/min), a longer ICU stay (5 vs.4 days) and included a dramatically higher proportion of patients with LV dysfunction (28% vs. 3%). The 2 groups had similar blood pressure, anterior wall location and management delays. Moreover, both groups were similar for CRP, eGFR and blood glucose levels. Interestingly, patients who developed symptomatic AF had a significantly higher level of ADMA (0.53 vs. 0.43 μmol/L), while SDMA and L-arginine were similar in the two groups. The rate of AF also showed a gradual increase across ADMA tertiles (21%, 55% and 74%, respectively, p<0.001) (Fig 1).

**Table 2:** Patients’ characteristics in the matched population (n = 58) (n(%), median (IQR) or mean±SD).

	No AF n = 29	Symptomatic AF n = 29	p
**Risk factors**			
Age, *years*	82 [74–88]	82 [74–89]	0.739
Female	12 (41%)	11 (38%)	0.788
BMI, *kg/m^2^*	26 ± 4	27 ±5	0.258
Hypertension	21 (72%)	21 (72%)	1
Hypercholesterolemia	13 (45%)	12 (41%)	0.791
Family history of CAD	10 (35%)	6 (21%)	0.240
Diabetes	7 (24%)	6 (21%)	0.753
Smoking	3 (10%)	3 (10%)	1
**CV history**			
CAD	7 (24%)	11 (38%)	0.256
Stroke	1 (3%)	2 (7%)	1
Chronic renal failure	0 (0%)	5 (17%)	0.052
Atrial fibrillation	1 (3%)	6 (21%)	0.102
**Chronic medications**			
Aspirin	8 (28%)	10 (35%)	0.570
Beta-Blocker	11 (38%)	13 (45%)	0.594
ACE inhibitor	7 (24%)	10 (35%)	0.387
Diuretic	14 (48%)	9 (31%)	0.180
Statin	10 (35%)	12 (41%)	0.588
VKA	2 (7%)	3 (10%)	1
Digoxin	1 (3%)	1 (3%)	1
Amiodarone	1 (3%)	0 (0%)	1
**Acute therapies**			
Aspirin	28 (97%)	28 (97%)	1
Beta-Blocker	23 (79%)	17 (59%)	0.089
ACE inhibitor	15 (52%)	7 (24%)	0.030
Diuretic	9 (31%)	16 (55%)	0.063
Statin	29 (100%)	28 (97%)	1
VKA	1 (3%)	3 (10%)	0.611
Digoxin	1 (3%)	1 (3%)	1
Amiodarone	1 (3%)	13 (45%)	<0.001
**Clinical data**			
HR, *beats/min*	77 ± 13	90 ±18	0.002
SBP, *mmHg*	139 ± 28	130 ± 26	0.186
DBP, *mmHg*	76 ± 17	77 ± 16	0.928
Heart failure	9 (31%)	16 (55%)	0.063
Anterior wall location	9 (31%)	12 (41%)	0.412
STEMI	12 (41%)	10 (35%)	0.588
Symptom onset to admission, *min*	260 [82–354]	300 [148–735]	0.212
ICU stay, *days*	4 [3–5]	5 [4–7]	0.003
LVEF (%)	50[45–63]	40 [35–55]	0.002
LVEF<40%	1(3%)	8 (28%)	0.025
LA Volume indexed (mL/m²)	30.9[21.4–42.9]	33.9 [25.4–48.2]	0.260
**Biological data**			
CRP≥3, *mg/L*	17 (59%)	20 (69%)	0.412
eGFR *ml/min*	60.1 ± 24.9	56.8 ± 23.4	0.602
Glucose, *mmol/L*	7.12 ± 2.25	7.85 ± 2.57	0.257
Nt-proBNP, *pg/mL*	916(411–2592)	4929(1144–18412)	0.001
L-arginine, *μmol/L*	70.9 [56.0–95.6]	75.8 [54.5–91.5]	0.969
ADMA, *μmol/L*	0.43 [0.38–0.48]	0.53 [0.46–0.63]	0.001
SDMA, *μmol/L*	0.67 [0.47–0.78]	0.76 [0.51–1.00]	0.250

ACE: Angiotensin Converting Enzyme; ADMA: Asymmetric Dimethyl Arginine; AF: Atrial fibrillation; BMI: body mass index; CAD: Coronary artery disease; CKD: Chronic Kidney Disease-Epidemiology Collaboration; CRP: C-reactive protein; DBP: Diastolic blood pressure; eGFR-MDRD: Estimated Glomerular Filtration Rate- Modification of Diet in Renal Disease; HR: heart rate; ICU: Intensive Care Unit; LVEF: Left ventricular ejection fraction; Nt-proBNP: N-terminal fragment of B-type natriuretic peptide; SBP: Systolic blood pressure; SDMA: Symmetric Dimethyl Arginine; STEMI: ST-segment elevation Myocardial Infarction; VKA: Vitamin K Antagonist.

### Factors associated with symptomatic AF

Multivariate logistic regression analysis for AF risk assessment was performed (Table 3). A first model (model 1) included the variables univariately associated with AF, i.e. HR on admission, LVEF<40% and a history of AF. In the second model (model 2), ADMA was added as a covariate to model 1. ADMA was an independent predictor of AF (OR(95%)CI: 2.46(1.21–5.00)), with a significant incremental value (model 2 vs model 1: NRI = 0.966, p<0.001, -2LL comparison: p = 0.003, and Bayesian Information Criterion: 68.0 vs. 73.6).The quality of the 2 models to estimate AF risk was good (Hosmer-Lemeshow goodness-of-fit p = 0.711 and p = 0.510). By univariate analysis, Nt-proBNP was significantly associated with AF (OR(95%CI): 1.84(1.23–2.75), p = 0.003). Interaction analysis confirmed that this relation may be at least partly linked to impaired LV ejection fraction in AF groups (test for interaction Nt-proBNP * LVEF<40%, p = 0.034). Given this strong association, Nt-proBNP was not included as a covariate together with LVEF, to avoid multicollinearity. When added to the multivariate model instead of LVEF (model 2), ADMA remained significantly associated with AF (OR(95%CI): 2.19(1.09–4.00), p = 0.028).

**Table 3:** Multivariate logistic regression models to estimate symptomatic AF risk in the matched population (n = 58).

	Model 1			Model 2		
Variable	OR	95%CI	p	OR	95%CI	p
History of AF	5.48	0.56–54.23	0.145	8.83	0.60–130.00	0.112
LVEF<40%	5.83	0.61–55.51	0.125	8.07	0.68–95.06	0.097
HR admission, *per 10 beats/min*	1.60	1.09–2.36)	0.018	1.52	0.90–2.35	0.060
ADMA, *per 0*.*1* *μmol/L*	-	-	-	2.46	1.21–5.00	0.013
-2LL Model 2 vs. model 1: p = 0.003	54.5			63.5		
Bayesian Information Criterion	68.0			73.6		
P Hosmer-Lemeshow goodness of fit	0.711			0.510		

ADMA = Asymmetric dimethylargigine; AF: Atrial Fibrillation; HR: Heart Rate; LA: Left atrial; LL = LogLikelihood; LVEF: Left ventricular ejection fraction.

## Discussion

Our work provides important data on ADMA levels in high-risk patients such as patients with AF in AMI. Our main findings are as follows: 1) ADMA levels were higher in AF patients than non-AF patients. 2) ADMA levels were higher in symptomatic than in silent AF 3) ADMA levels estimated the risk of developing AF beyond traditional determinants of the arrhythmia.

### Association between dimethylarginine and AF

We found that SDMA levels, along with age, gradually increased across the 3 groups. SDMA does not appear to directly inhibit NO synthesis although it has been reported to competitively inhibit L-arginine uptake. Other authors have reported stimulating effects of SDMA on ROS production by monocytes [25] but further work in this area is clearly needed. SDMA is currently considered a marker of advanced age and renal disease [26]. In our study, after adjustment for age, SDMA did not remain an independent factor associated with the risk of symptomatic AF after AMI.

Our findings are in accordance with recent data showing that ADMA levels are higher in age-matched patients with AF than in control non-AF patients [18, 19]. ADMA levels are elevated in both acute and persistent episodes of tachyarrhythmia or after prolonged rapid atrial pacing (i.e.7 hours) [18]. Moreover, increased serum ADMA concentrations are associated with a higher risk of recurrence after catheter ablation or electric cardioversion [27, 28]. Our results showed for the first time a significant association between ADMA levels and symptomatic AF occurrence after AMI, after adjustment for age. When compared with patients without AF, ADMA was elevated only in symptomatic AF. As AF patients have more frequently heart failure, the levels of ADMA could be linked to AF through impaired LV function. Oxidative stress, as an excess production of ROS relative to antioxidant defense, has been shown to play an important role in the underlying mechanisms of cardiac remodeling and HF, through changes in intracellular pathways, redox signaling, at lower levels, and causes cardiomyocyte dysfunction and damage at higher levels. ADMA might increase oxidative stress by uncoupling of the electron transport between NO synthase and L-arginine, which can lead to decreases in NO bioavailability. Elevated ADMA levels were reported in patients with congestive HF, and correlated significantly with New York Heart Association (NYHA) functional class and exercise capacity [29]. In addition, ADMA levels are associated with adverse cardiovascular outcome in patients with chronic HF [30, 31].

However, ADMA level was not significantly increased in patients with silent AF, in spite of reduced ejection fraction. Moreover, ADMA remains an independent estimate of AF beyond LV impairment (Table 3). This could indicate that only a marked elevation of ADMA may promote AF episodes and at least partly independently of LV dysfunction.

ADMA is a known endogenous eNOS inhibitor and a risk factor for endothelial dysfunction [32]. ADMA has cytokine-like properties and profoundly impairs NO synthesis, thus resulting in polymorphonuclear neutrophil activation and adhesion to endothelial cells [33]. ADMA also promotes vascular Reactive Oxygen Species (ROS) production via eNOS uncoupling in patients with advanced atherosclerosis [7, 34]. Although ADMA itself does not reduce eNOS gene expression in human endothelial cell cultures, altered NO availability may favor the development of AF, because of abnormalities in Ca^2+^ handling and fibrosis stimulation [18]. Moreover, a causative link between oxidative stress and AF has recently been demonstrated in human tissues, with NADPH oxidases as a source of ROS [35, 36]. Experimental data showed that AF causes a down-regulation of NOS expression, with a fall in NO concentrations of 73% in the left atrial appendage [37], suggesting a positive feedback loop in the maintenance of AF. High ventricular rates associated with AF may impair hemodynamics in AMI patients by increasing oxygen demand, a situation that may be aggravated by the microcirculatory effects of elevated levels of ADMA. Furthermore, by reducing endothelial NO production, ADMA accumulation may result in endothelial dysfunction in the LA, thus promoting thrombus formation and subsequent thromboembolic events [38]. Unfortunately, in our study the rate of stroke was too low (n = 1) to analyse this outcome. Further experimental studies are needed to fully elucidate the underlying mechanisms linking ADMA and AF occurrence.

### ADMA to predict AF occurrence

Our findings on the clinical characteristics of AF in AMI are in accordance with most studies, which reported advanced age, heart failure, elevated HR, and depressed LV function, as predictors of AF and even in contemporary reperfusion strategies [3]. In patients with chronic heart failure, ADMA levels were associated with advanced systolic dysfunction and independently predicted overall long-term adverse event rates [39]. In our study, ADMA was an independent predictor of AF, beyond LV dysfunction, strongly suggesting that measuring ADMA levels at baseline could improve risk prediction for symptomatic AF in the setting of AMI. Therefore, ADMA level, as a marker of endothelial oxidative stress or impaired NOS activity, provides additional prognostic information, complementary to LV dysfunction or heart failure symptoms, which may be particularly relevant in these high-risk patients.

### Study limitation

Due to the small sample size, it was not possible to analyze the thromboembolic event rate (One stroke occurred during the hospital stay). Larger studies are needed to investigate the relationship between AF, ADMA and ischemic events. Although it could be interesting to address the relationship between serum levels of ADMA and the number of AF episodes, the duration of the silent AF episodes could not be established using our recording methods. Moreover, this work suffered from the usual limitations of observational, non-randomized studies and therefore determined correlations rather than causal relationships.

## Conclusion

Although the occurrence of AF in AMI is common and carries a very high risk, the underlying mechanisms are not fully understood. Our prospective study showed for the first time that the occurrence of AF was associated with ADMA, a circulating marker related to endothelial dysfunction and oxidative stress. Although no causative role can be concluded from our observational study, our work further supports the hypothesis that oxidative stress is involved in the pathogenesis of AF in AMI. Moreover, our works strongly suggests that baseline levels of ADMA could improve risk stratification in this high-risk group, beyond older age and LV dysfunction.
